# Association between dispensing regulations for extended-release methylphenidate and prescription trends in Japan: an exploratory descriptive study

**DOI:** 10.1186/s40780-025-00531-5

**Published:** 2025-12-22

**Authors:** Kazumasa Kotake, Toshiki Nakamura, Yuki Nakano, Naoya Kitamura

**Affiliations:** 1 Department of Pharmacy, Zikei Hospital, Zikei Institute of Psychiatry, 100-2 Urayasu Honmachi, Minami-Ku, Okayama-shi, Okayama, 702-8508 Japan; 2 Akebono Pharmacy Group, Akebono Pharmacy, 1-3-1 Amakubo, Tsukuba-shi, Ibaraki 305-0005 Japan; 3 Department of Pharmacy, Saga University Hospital, 5-1-1 Nabeshima, Saga, 849-8501 Japan; 4 Department of Neuropsychiatry, Okayama Saiseikai General Hospital, 2-25 Kokutaicho, Kita-ku, Okayama- shi, Okayama, 700-8511 Japan

**Keywords:** Attention deficit hyperactivity disorder, Extended-release methylphenidate, Neurodevelopmental condition, Distribution-control policy

## Abstract

**Background:**

In December 2019, a nationwide registration system with strict distribution controls for extended-release methylphenidate (ER-MPH) was introduced to prevent inappropriate use while maintaining treatment access for patients with attention deficit hyperactivity disorder (ADHD). However, its impact on prescription patterns remains unclear.

**Methods:**

We conducted an observational study using Japan’s National Database of Health Insurance Claims from 2016 to 2022. ER-MPH prescription volumes were calculated as defined daily doses per 1,000 individuals. Interrupted time series (ITS) analysis was performed to assess the impact of the 2019 distribution-control policy across age and sex groups. We additionally examined whether regional differences in prescription trends could be explained by access to care by assessing the correlation between ER-MPH prescription volumes and the distribution of board-certified pediatricians using Pearson’s correlation coefficients.

**Results:**

ER-MPH prescriptions continued to increase after the 2019 policy implementation, but ITS analysis revealed a substantial slowdown in the growth rate. Prescriptions declined immediately by 7% at policy implementation **(**level change incidence rate ratio [IRR] = 0.93, 95% confidence interval [CI]: 0.89–0.96), and the annual growth rate slowed from 27% pre-policy (IRR = 1.27, 95% CI: 1.25–1.28) to 9% post-policy (IRR = 1.09, 95% CI: 1.08–1.10) (slope change IRR = 0.86, 95% CI: 0.85–0.87). Age-stratified analyses revealed differential impacts: individuals aged < 20 years showed a 47% immediate decline (IRR = 0.53, 95% CI: 0.42–0.68) with nearly flat subsequent growth, while adults aged ≥ 20 years had only a 19% reduction (IRR = 0.81, 95% CI: 0.79–0.84) and continued slower growth. Sex-specific analyses also showed attenuated growth. The correlation between prescription volumes and pediatrician distribution was weak, indicating no substantial relationship.

**Conclusions:**

The 2019 distribution-control measures markedly attenuated the existing upward trend, especially among individuals aged < 20 years. Although prescription patterns differed by age and sex, these differences alone did not fully explain the overall attenuation, and regional variation showed only weak correlations with pediatrician distribution. These findings suggest that additional factors such as psychiatrist availability and broader healthcare system characteristics may also have influenced prescribing trends, underscoring the need for further investigation.

## Background

 Attention deficit hyperactivity disorder (ADHD) is a prevalent neurodevelopmental condition that affects both children and adults [1]. Epidemiological data from Japan, derived from the National Database of Health Insurance Claims and Specific Health Checkups of Japan (NDB), demonstrated a marked increase in the incidence of newly diagnosed ADHD cases between 2010 and 2019 across all age groups [2]. This rising prevalence is of particular concern because individuals with ADHD often experience substantial impairments in academic achievement [3], occupational performance [4], and social relationships [5]. ADHD treatment is categorized into pharmacological and non-pharmacological approaches [6]. Stimulant-based pharmacological treatment plays a central role in managing core symptoms and has demonstrated long-term benefits in symptom control [7–9].

The U.S. Food and Drug Administration approved immediate-release methylphenidate (IR-MPH) in 1955 for the treatment of ADHD [10]. In Japan, IR-MPH was introduced in 1958 for the treatment of narcolepsy and its use in ADHD treatment had become more common in the subsequent years. Owing to growing societal concerns regarding misuse and dependence [11], regulatory authorities implemented restrictions on IR-MPH use for ADHD in 2007 [12]. However, the extended-release methylphenidate (ER-MPH) was subsequently approved [13], and following regulatory actions, ER-MPH gradually replaced IR-MPH as the primary treatment option for ADHD in Japan. In December 2019, a nationwide registration system for ER-MPH was implemented to prevent inappropriate use, requiring official registration for prescribing physicians and dispensing pharmacies [14]. Although the government-mandated registration system aims to enhance the safety and traceability of ADHD medications, its effect on treatment access remains uncertain. By restricting the rights of prescribing medicines to certified specialists and requiring patient and pharmacist registration, the system may have unintentionally limited access.

Understanding the consequences of this regulatory shift is essential, as it may have curbed misuse and unintentionally restricted access to treatment. To assess these effects, we analyzed national trends in ER-MPH prescriptions using NDB Open Data, focusing on variations in age and sex, and examined their correlation with the regional distribution of board-certified pediatricians.

## Methods

### Data source

We conducted an observational study using the NDB, a comprehensive database of administrative claims maintained by the Ministry of Health, Labour and Welfare of Japan [15]. For this analysis, we specifically used NDB Open Data, a publicly accessible subset of the NDB. NDB Open Data have been widely used in clinical and epidemiological research in Japan [16, 17]. NDB Open Data consist of anonymized, publicly accessible information regarding prescription records and specific health checkups, with no personally identifiable data. Ethical approval or informed consent was not required for this study.

### Drug information

The NDB Open Data includes data on the top 100 pharmaceutical formulations by prescription volume within each pharmacological class, as defined by the Japanese Standard Commodity Classification issued by the Ministry of Internal Affairs and Communications [15]. We extracted data on the number of prescriptions (e.g., the number of tablets dispensed) for ER-MPH by age, sex, and prefectures. Japan is composed of 47 prefectures that serve as the primary administrative divisions and are equivalent to states or provinces in other countries [18]. We analyzed the prescription volumes at the prefectural level, which also allowed us to examine their correlation with the distribution of board-certified pediatricians, who hold prescribing authority for ER-MPH. The data cover the period from 2016 to 2022, which is 3 years before and after 2019, when stricter distribution controls were introduced under Japan’s ADHD Appropriate Use and Distribution Management System [14]. Because the policy was implemented in December 2019, we excluded calendar year 2019 from all analyses to avoid partial exposure within the implementation year. Consequently, the pre-policy period was defined as 2016–2018 and the post-policy period as 2020–2022 (three years before and after the policy introduction, respectively). We set 2016 as the first year of the study period because ER-MPH was approved for adult ADHD in December 2013 [15], and NDB Open Data are released as annual tabulations with a time lag [19]. Lisdexamfetamine was approved in Japan in 2019 and, similar to ER-MPH, has been subjected to a distribution control system aimed at preventing inappropriate use [20]. However, because the control system was implemented during its market launch, the data before and after the introduction of the system could not be compared. Therefore, lisdexamfetamine was excluded from this study.

### Calculation of prescription volume

To quantify the annual use of ER-MPH over the study period, we calculated the prescription volume using the number of dispensed tablets reported in the NDB Open Data. Drug utilization was expressed as defined daily doses (DDD) per 1,000 individuals (DID), based on the methodology recommended by the World Health Organization (WHO). According to the WHO Collaborating Centre for Drug Statistics Methodology, 1 DDD of methylphenidate (Anatomical Therapeutic Chemical code: N06BA04) corresponds to 30 mg [21]. The DID was calculated using the following formula based on extant studies [21, 22]:

$${\text{DID = }}\frac{\begin{gathered}({\text{number of tablets dispensed }} \hfill \\\times {\text{ dose per tablet}}) \hfill \\ \end{gathered} }{{({\text{DDD }} \times {\text{ 365 }} \times {\text{ population}})}} \times {\text{ 1,000}}$$

This formula was applied separately for each year using the annual tablet count, dosage strength, and corresponding population size to compare ER-MPH utilization trends year-by-year. Additionally, to examine ER-MPH use according to demographic factors, we calculated the prescription volumes stratified by age and sex.

### Statistical analysis

To evaluate the impact of the distribution-control policy introduced in December 2019, we conducted an interrupted time series (ITS) analysis. A generalized linear model with a negative binomial distribution was fitted, with the annual DID of ER-MPH as the dependent variable and the logarithm of the population size as an offset. This model estimated level and slope changes before and after the policy implementation, expressed as incidence rate ratios (IRRs) with 95% confidence intervals (CIs). Residual autocorrelation was to be assessed using the autocorrelation function, the partial autocorrelation function, and the Ljung–Box test. However, given the extremely limited number of time points, these assessments were not feasible, and no formal adjustment for autocorrelation was applied.

Analyses were also conducted by sex and by age using aged < 20 years as the cutoff, because previous studies have shown that prescription trends differ substantially between children/adolescents and adults [22], and also by sex.

Given that the attenuation of prescription trends was most pronounced among individuals aged < 20 years, we hypothesized that the distribution of board-certified pediatricians—who are primarily responsible for prescribing ER-MPH in this age group—could influence this finding. Therefore, we examined the correlation between the DID of ER-MPH and the number of board-certified pediatricians per 100,000 population across all 47 prefectures. As physician statistics in Japan are updated biennially, we used the available data for the representative years before and after the policy implementation (2016, 2018, 2020, and 2022). Pearson’s correlation coefficients were calculated separately for each year.

All analyses were conducted using R version 4.2.0. ITS analysis was performed with the tsModel package and data manipulation and visualization using the tidyverse (version 2.0.0) and ggplot2 (version 3.5.1) packages.

## Results

### Prescription trends for ER-MPH before and after policy implementation

**Fig. 1:**
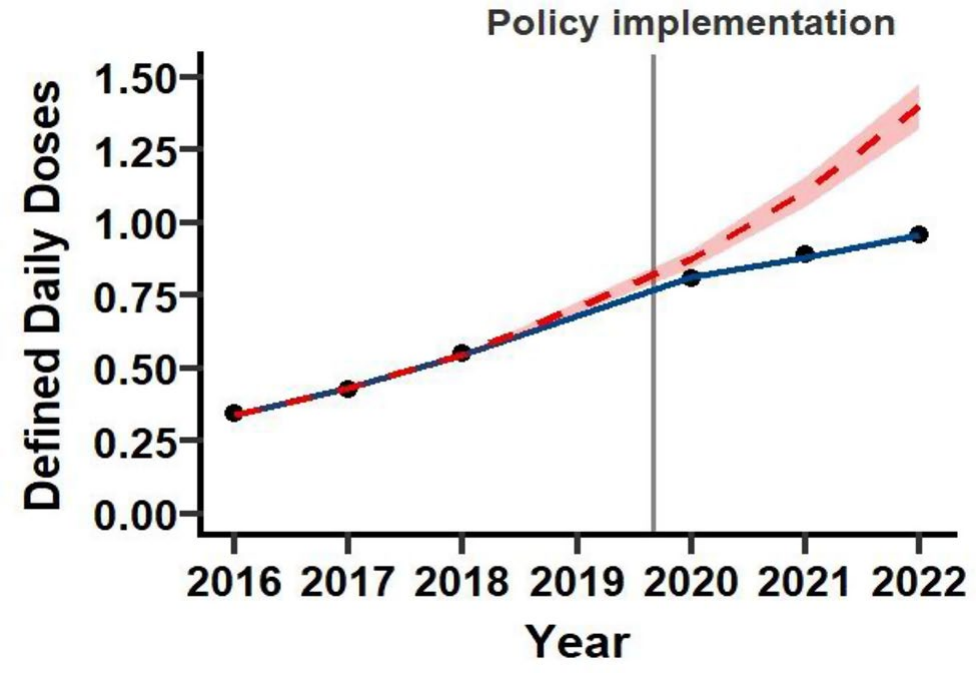
Prescription trends for ER-MPH before and after policy implementation. The solid blue line represents the model-fitted trend for defined daily doses per 1,000 individuals (DID) with corresponding 95% confidence intervals (blue shaded area). The dashed red line indicates the counterfactual prediction (i.e., the estimated prescription trend if the policy had not been implemented), with its 95% confidence intervals (red shaded area). The black circles represent the observed annual DIDs. The gray vertical line marks the timing of the policy implementation in December 2019. Although the x-axis is labeled “Year,” it corresponds to the Japanese academic year, which runs from April to March

The prescription trends for ER-MPH are shown in Fig. 1. The ITS analysis indicated a level change of IRR = 0.93 (95% CI: 0.89–0.96) and a slope change of IRR = 0.86 (95% CI: 0.85–0.87) at policy implementation. The pre-policy slope was IRR = 1.27 (95% CI: 1.25–1.28), compared with IRR = 1.09 (95% CI: 1.08–1.10) after implementation, indicating substantial attenuation of the increasing trend.

### Prescription trends for ER-MPH by age and sex before and after policy implementation

**Fig. 2:**
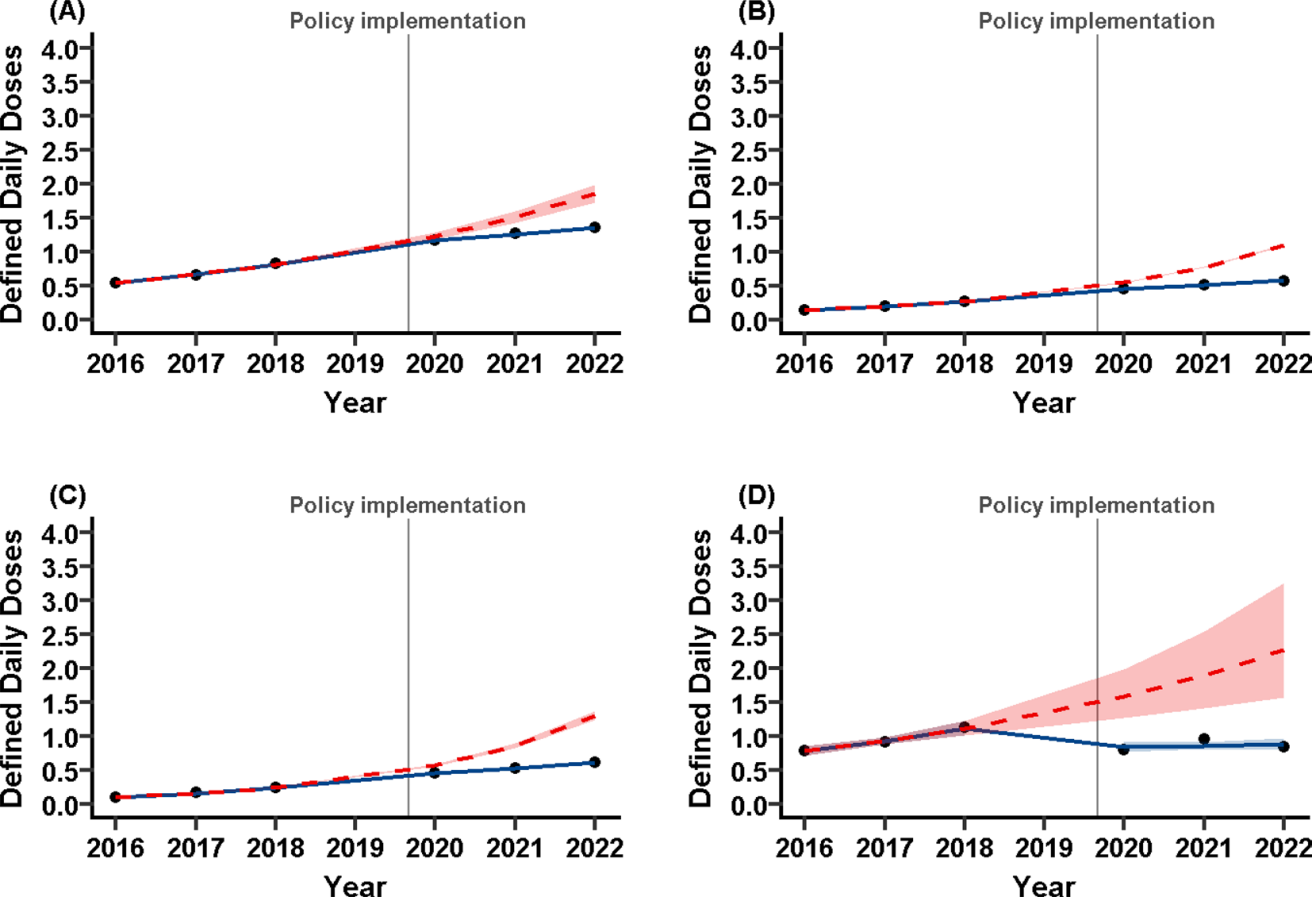
Prescription trends of ER-MPH by age and sex before and after policy implementation. (**A**) Male, (**B**) Female, (**C**) Age ≥ 20 years, and (**D**) Age < 20 years. The black circles represent the observed data, expressed as defined daily doses per 1,000 individuals (DID). The solid blue line represents the model-fitted trend, with the surrounding blue shaded area indicating the 95% confidence interval. The dashed red line represents the counterfactual trend (i.e., the expected prescription trend if the policy had not been implemented), with the surrounding red shaded area indicating the 95% confidence interval. The gray vertical line marks the timing of the policy implementation in December 2019. Although the x-axis is labeled “Year,” it corresponds to the Japanese academic year, which runs from April to March

To explore the possible reasons for the attenuation of the increasing trend, Fig. 2 presents the analyses by age and sex. Among males, the level change was IRR = 0.95 (95% CI: 0.91–0.99) and the slope change IRR = 0.88 (95% CI: 0.86–0.90), with the slope decreasing from IRR = 1.23 (95% CI: 1.21–1.24) before the policy to 1.08 (95% CI: 1.06–1.09) after; this indicates a moderated but still increasing trend. Among females, the level change was IRR = 0.83 (95% CI: 0.82–0.84) and the slope change IRR = 0.80 (95% CI: 0.80–0.80), with the slope declining from IRR = 1.40 (95% CI: 1.40–1.41) to 1.13 (95% CI: 1.12–1.13), again showing an attenuated but persistent increase. In adults aged ≥ 20 years, the level change was IRR = 0.81 (95% CI: 0.79–0.84) and the slope change IRR = 0.76 (95% CI: 0.75–0.77), with the slope reduced from IRR = 1.51 (95% CI: 1.49–1.52) to 1.15 (95% CI: 1.14–1.16), indicating continued growth albeit slower. By contrast, in individuals aged < 20 years, the level change was IRR = 0.53 (95% CI: 0.42–0.68) and the slope change IRR = 0.86 (95% CI: 0.77–0.95), with the slope flattening from IRR = 1.19 (95% CI: 1.11–1.28) to 1.02 (95% CI: 0.95–1.10), showing that the upward trend nearly disappeared. Altogether, these results indicate that although the distribution-control policy moderated growth in all subgroups, the most pronounced attenuation occurred among individuals aged < 20 years.

### Prescription trends for ER-MPH among individuals aged < 20 years by sex

**Fig. 3:**
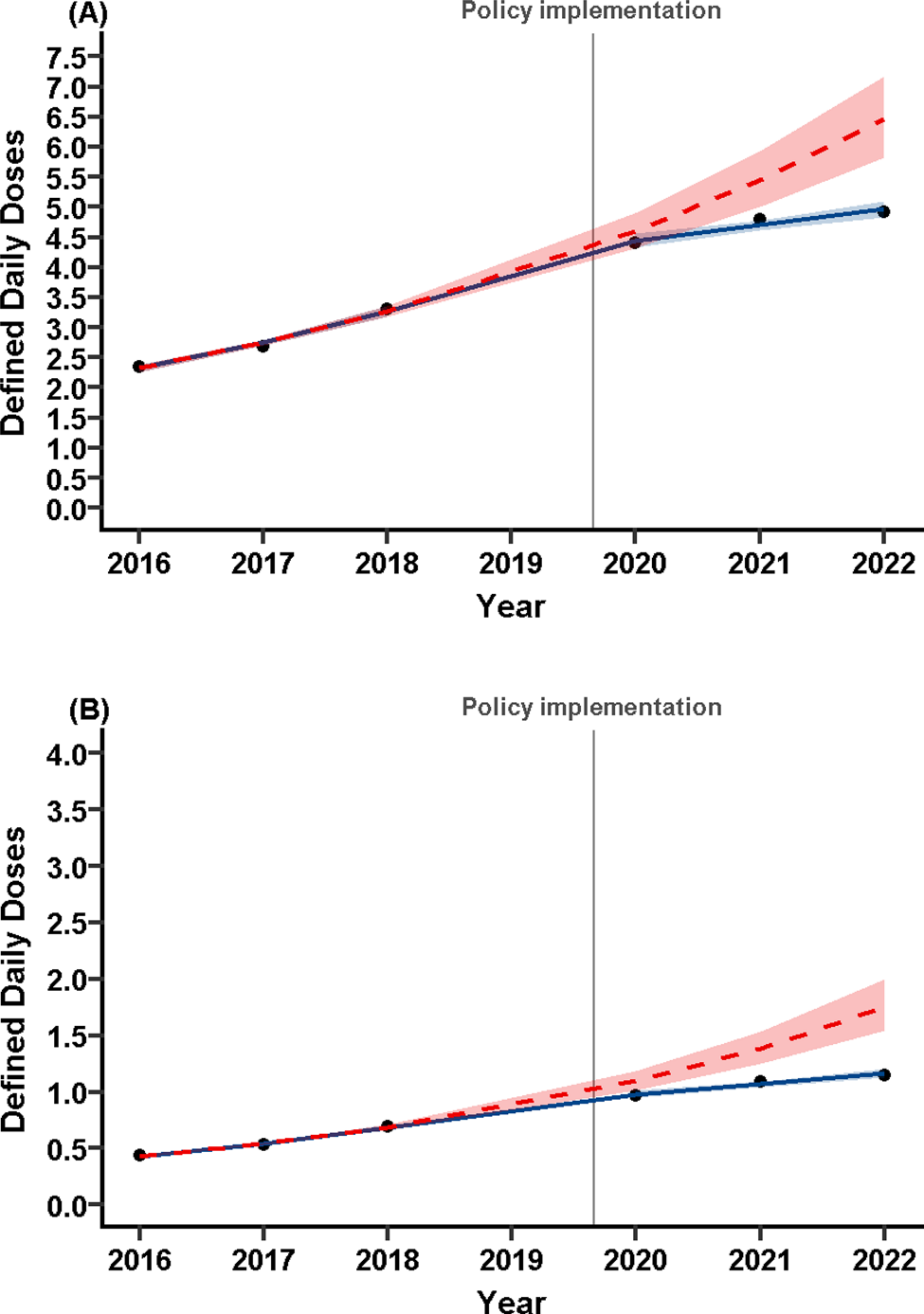
Prescription Trends of ER-MPH among Individuals Aged < 20 Years by Sex. Interrupted time series (ITS) analyses stratified by sex are shown for (**A**) males and (**B**) females < 20 years of age. The blue solid line represents the observed defined daily doses (DDD) per 1,000 individuals per day (DID). The red dashed line with shaded 95% confidence interval represents the predicted counterfactual trend in the absence of the 2019 policy implementation (shaded area). The gray vertical line indicates the timing of the policy implementation in December 2019. Although the x-axis is labeled “Year,” it corresponds to the Japanese academic year (April to March)

Because Fig. 2 shows that the attenuation of the increasing trend was most pronounced among individuals aged < 20 years, we examined whether this pattern differed by sex. Figure 3 presents the ITS results for those aged < 20 years.

Among males (Fig. 3A), the level change at policy implementation was not significant (IRR = 0.97, 95% CI: 0.90–1.04), whereas the slope change was IRR = 0.89 (95% CI: 0.87–0.92). The slope decreased from IRR = 1.19 (95% CI: 1.16–1.21) before the policy to 1.06 (95% CI: 1.04–1.08) after, indicating that the increasing trend was attenuated but persisted.

Among females (Fig. 3B), both the level change (IRR = 0.89, 95% CI: 0.82–0.97) and slope change (IRR = 0.86, 95% CI: 0.83–0.89) were significant. The slope declined from IRR = 1.26 (95% CI: 1.23–1.30) to 1.09 (95% CI: 1.06–1.12), showing a modest immediate decline followed by a slower but still upward trend.

Altogether, these results indicate that the policy substantially moderated ER-MPH prescription growth among both sexes for those aged < 20 years.

### Correlation between pediatrician density and ER-MPH prescriptions

Because the attenuation of prescription trends was most pronounced among individuals aged < 20 years, we focused on board-certified pediatricians, who are primarily responsible for prescribing ER-MPH in this age group, and examined their potential contribution to regional variation. Specifically, we analyzed the correlation between the number of board-certified pediatricians per 100,000 population and the DID of ER-MPH across prefectures in representative years before and after the policy implementation (2016, 2018, 2020, and 2022).

**Fig. 4:**
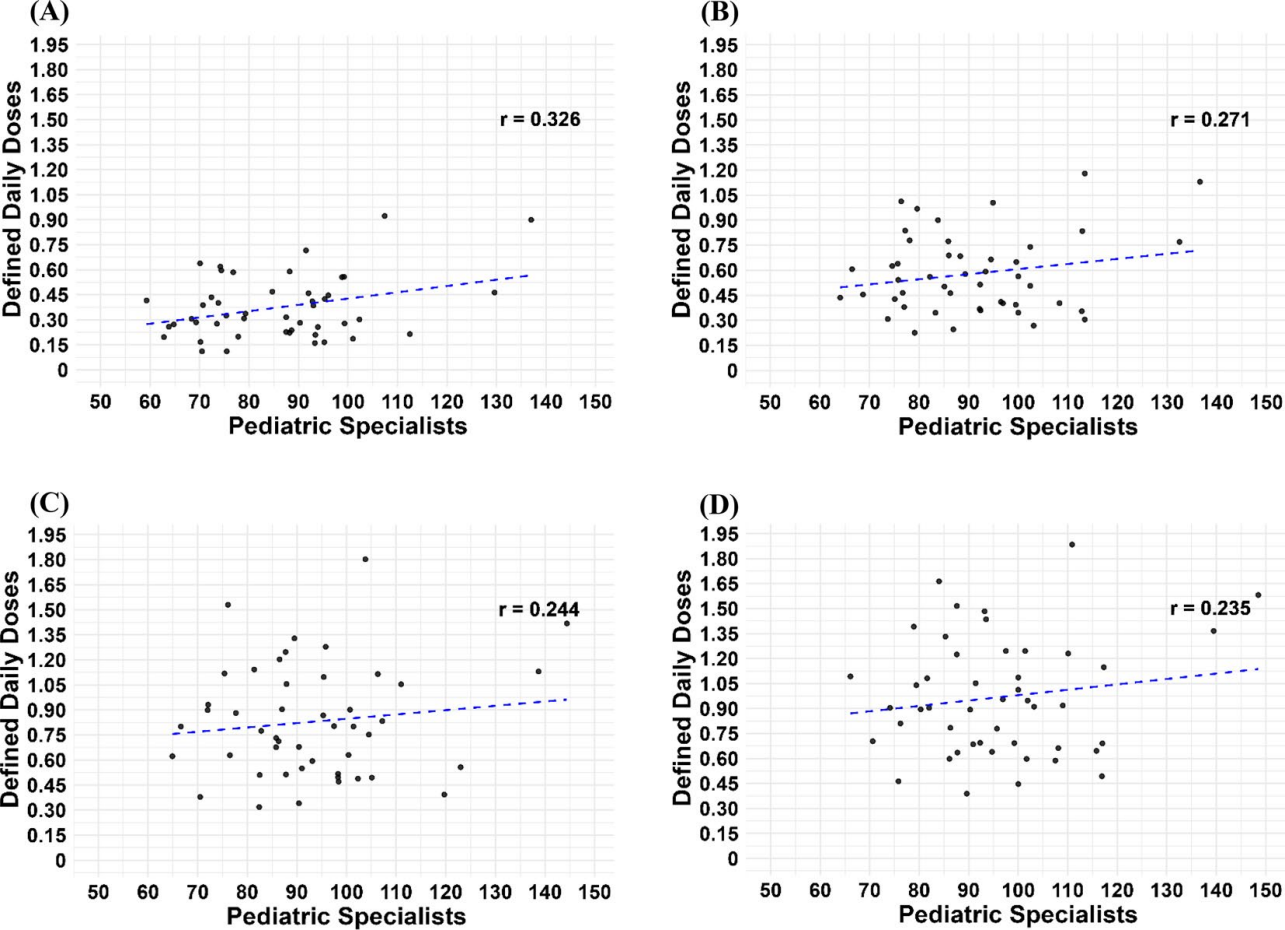
Correlation between pediatrician density and ER-MPH prescriptions across prefectures in Japan. Scatter plots display the relationship between the number of board-certified pediatricians per 100,000 population and the defined daily doses (DID) of extended-release methylphenidate (ER-MPH) across the 47 prefectures. Each panel represents a different year: (**A**) 2016, (**B**) 2018, (**C**) 2020, and (**D**) 2022. The blue dashed lines indicate the fitted regression line; correlation coefficients (Pearson’s r) are shown in each panel

As shown in Fig. 4, weak positive correlations were observed in all years (2016: *r* = 0.326; 2018: *r* = 0.271; 2020: *r* = 0.244; 2022: *r* = 0.235). Although the coefficients gradually decreased after the policy implementation, they consistently remained within the range of weak correlations.

## Discussion

Our study showed that the 2019 distribution-control policy significantly attenuated the upward trend in ER-MPH prescriptions in Japan, with the effect being most pronounced among individuals aged < 20 years. We then examined whether this attenuation correlated with pediatrician density. However, only weak correlations between the attenuation and pediatrician density were observed, suggesting that prescriber distribution alone does not fully explain the observed attenuation. While these findings indicate that the policy had a measurable impact on prescription trends, other factors—such as the coronavirus disease 2019 (COVID-19) pandemic and the distribution of psychiatrists authorized to prescribe ER-MPH—may also have influenced trends. Therefore, further investigation is needed to clarify the relative contributions of these factors.

The ITS analysis demonstrated that the introduction of the distribution-control policy significantly attenuated the upward trend in ER-MPH prescriptions (level change IRR = 0.93, slope change IRR = 0.86), but the overall increasing trend was not reversed. Studies have examined temporal trends in ADHD-medication prescriptions and the use of stimulant and non-stimulant ADHD drugs, conducted a total population analysis of trends and discontinuation patterns during 2006–2009 [21, 23], and examined changes in the prescribing patterns following media coverage [24] and the effects of age-based prescription restrictions [25]. However, to the best of our knowledge, no prior research has evaluated the impact of implementing a distribution control system.

Sex-specific analyses revealed differential impacts of the distribution-control policy on males and females. Among males, the level change at policy implementation was not statistically significant (IRR = 0.97, 95% CI: 0.90–1.04, *p* = 0.30), whereas the slope change was significant (IRR = 0.89, 95% CI: 0.87–0.92, *p* < 0.001). This indicated that the upward prescription trend persisted but became more moderate. By contrast, females showed significant changes in both level (IRR = 0.89, 95% CI: 0.82–0.97, *p* < 0.001) and slope (IRR = 0.86, 95% CI: 0.83–0.89, *p* < 0.001), indicating an immediate decline followed by markedly slower subsequent growth. Females with ADHD have historically been under-diagnosed and under-treated, leading to a smaller population receiving pharmacological treatment [26]. When the treated population is small, external shocks can produce proportionally larger fluctuations in observed prescribing patterns. Thus, the more pronounced attenuation among females may partly reflect the heightened susceptibility of this limited treated group to system-level disruptions. Moreover, the timing of the policy coincided with the onset of the COVID-19 pandemic, during which disruptions in healthcare utilization and ADHD-related prescriptions were widely reported [27–29], potentially amplifying these fluctuations.

Regarding the prescription trends by age, clear differences emerged. In adults aged ≥ 20 years, both the level (IRR = 0.81, 95% CI: 0.79–0.84) and slope change (IRR = 0.76, 95% CI: 0.75–0.77) were significant, indicating that prescriptions continued to increase but at a slower pace after the policy. By contrast, in individuals aged < 20 years, the level change was markedly lower (IRR = 0.53, 95% CI: 0.42–0.68), and the post-policy slope (IRR = 1.02, 95% CI: 0.95–1.10) was no longer significantly different from zero, suggesting that the increasing trend almost disappeared. These findings indicate that the policy had a stronger influence on those aged < 20 years, potentially due to differences in prescribing authority and access to specialized care. In addition, external factors such as the COVID-19 pandemic, which reduced opportunities for medical visits and new diagnoses [28, 30], may have disproportionately affected younger populations, who are more reliant on specialist access for ADHD management. Further stratification of the < 20 group by sex confirmed this overall pattern. Although males showed attenuation mainly in the slope and females in both the level and slope, both sexes consistently exhibited a slowing of prescription growth. Thus, the marked attenuation observed among individuals aged < 20 years cannot be attributed to sex differences but rather reflects the broader impact of the policy and contextual factors.

To further investigate the pronounced attenuation observed in individuals aged < 20 years, we examined the potential effect of pediatrician availability, as board-certified pediatricians are primarily responsible for prescribing ER-MPH in this age group. However, the correlation between pediatrician density and ER-MPH prescription volume remained weak and showed little change both before and after the policy (2016: *r* = 0.326; 2018: *r* = 0.271; 2020: *r* = 0.244; 2022: *r* = 0.235). These findings suggest that pediatrician distribution alone does not fully explain the observed attenuation. Instead, broader contextual factors—such as the introduction of the distribution-control system, disruptions in healthcare utilization during the COVID-19 pandemic, or other regional healthcare resource disparities—may have played a larger role in shaping prescription trends.

This study has some limitations that should be considered when interpreting the findings. First, this study utilized the NDB Open Data, which are aggregated and anonymized, preventing analysis at the individual patient level. Therefore, we could neither distinguish between newly initiated and continuing ER-MPH users nor assess clinical appropriateness, treatment adherence, or treatment outcomes. Second, while ER-MPH can be prescribed by both pediatricians and psychiatrists, prefecture-level data on psychiatrists with prescribing authority were not available. Therefore, we used pediatrician density as a proxy for prescriber availability. However, the weak correlation observed suggests that pediatrician distribution alone does not adequately capture regional variation, highlighting the need for future studies incorporating data on psychiatrists and other qualified prescribers. Third, as this was an ecological study, causality could not be inferred. The observed associations reflect population-level trends and may not directly translate to individual-level treatment access or decision making. Fourth, the ITS analysis included a limited number of pre- and post-policy time points. Although the level and slope changes were clearly detected in our analysis, studies with fewer observations may yield somewhat less stable estimates, and therefore some caution is warranted when interpreting the magnitude of these changes [31]. Finally, while this study focused on prescriber-related factors, the potential burden on dispensing pharmacies should be considered. Under the 2019 distribution-control system, both prescribers and pharmacies are required to register, which may have introduced an additional administrative workload and affected patient access. To overcome these limitations, future studies should incorporate individual-level data, include other relevant prescribers such as psychiatrists, extend the observation period to improve the precision of trend estimates, and conduct more detailed analyses at the prefectural level.

Despite these limitations, the major strength of this study lies in its use of a comprehensive nationwide claims database, which enabled robust analyses of ER-MPH prescription patterns by age, sex, and region both before and after policy implementation.

## Conclusions

Despite the nationwide increase in ER-MPH prescriptions, the implementation of strict distribution-control measures in 2019 significantly attenuated this upward trend, particularly among individuals < 20 years of age. When regional variation was examined, only weak correlations with pediatrician density were observed, suggesting that prescriber distribution alone does not account for the observed differences. These findings highlight the importance of further investigations of additional factors, including the role of psychiatrists and broader healthcare system characteristics, to ensure equitable access to ADHD treatment.

## Data Availability

The data supporting the findings of this study are publicly available from the National Database of Health Insurance Claims and Specific Health Checkups of Japan (NDB Open Data), published by the Ministry of Health, Labour and Welfare (https://www.mhlw.go.jp/stf/seisakunitsuite/bunya/0000177182.html). Additional data are available from the first author upon request ( [*kottanketty@gmail.com)*](.) .

## Abbreviations

ADHD: Attention deficit hyperactivity disorder
DDD: Defined daily doses
DID: Defined daily doses per 1,000 individuals
ER-MPH: The extended-release methylphenidate
IR-MPH: The immediate-release methylphenidate
NDB: National Database of Health Insurance Claims
ITS: Interrupted Time Series
IRR: Incidence Rate Ratio
CI: Confidence Interval
WHO: World Health Organization
MHLW: Ministry of Health, Labour and Welfare of Japan
FDA: Food and Drug Administration
COVID-19: Coronavirus Disease 2019
